# Clinical safety and efficacy of bispecific antibody in the treatment of solid tumors: A protocol for a systematic review

**DOI:** 10.1371/journal.pone.0271506

**Published:** 2022-07-18

**Authors:** Seyed Aria Nejadghaderi, Maryam Balibegloo, Amene Saghazadeh, Nima Rezaei

**Affiliations:** 1 Systematic Review and Meta-Analysis Expert Group (SRMEG), Universal Scientific Education and Research Network (USERN), Tehran, Iran; 2 Cancer Immunology Project (CIP), Universal Scientific Education and Research Network (USERN), Tehran, Iran; 3 Network of Immunity in Infection, Malignancy and Autoimmunity (NIIMA), Universal Scientific Education and Research Network (USERN), Tehran, Iran; 4 Research Center for Immunodeficiencies, Children’s Medical Center, Tehran University of Medical Sciences (TUMS), Tehran, Iran; 5 Department of Immunology, School of Medicine, Tehran University of Medical Sciences (TUMS), Tehran, Iran; Public Library of Science, UNITED KINGDOM

## Abstract

**Background:**

Cancers are among the most common causes of mortality and morbidity. Recently, bispecific antibodies (BsAbs) have been used for cancer treatment. The aim of this systematic review and meta-analysis will be to determine the safety and efficacy of BsAbs in the treatment of solid tumors.

**Methods:**

We will search five electronic databases, PubMed, EMBASE, Scopus, Web of Science, and CENTRAL, in addition to Clinical-Trials.gov and metaRegister of controlled trials and backward and forward citation searching of included studies. Eligible studies will be controlled clinical trials evaluating safety and/or efficacy of BsAbs in adult patients with solid tumors. The primary outcomes will be the incidence of safety and efficacy measures. Title and/or abstract screening, full text reviewing, data collection, and quality assessment will be done by two reviewers. We will use The Cochrane Collaboration’s risk of bias tool 2 (RoB2) to assess the quality of included studies. If I-square heterogeneity was greater than 40%, we will implement random effect model. Subgroup analysis and meta-regression will be undertaken if applicable. The metaprop command of STATA will be used to calculate frequency of AEs. Funnel plot, Egger’s and Peter’s tests will be utilized to evaluate publication bias in case of including at least ten studies. We will use sensitivity analysis to evaluate the effects of funding sources and continuity correction on effects size.

**Conclusions:**

The findings of the present study will provide information on safety and efficacy of BsAbs for physicians and researchers in the management of solid tumors.

**Trial registration:**

Registration on PROSPERO CRD42021227879 Also, important protocol amendments will be stated on PROSPERO registration.

## Introduction

Among noncommunicable diseases which are the leading cause of death in the world, cancer is one of the great challenges in health-related issues, with estimated 19.3 million new cases and 10.0 million deaths globally in 2020 [1]. In addition, the Global Cancer Incidence, Mortality and Prevalence (GLOBOCAN) 2020 findings showed female breast cancer, lung cancer, and colorectal cancer had the highest incidence and lung, colorectal, and liver cancers had the greatest mortality in both sexes around the world in 2020 [1]. The attributable disability-adjusted life years (DALYs) of solid cancers were 249.0 million in 2019 worldwide [2]. In 2019, mortality and incidence of cancers were higher in males in the world, age-standardized incidence rate of 348.7 in males versus 246.1 in females per 100,000 people, and age-standardized mortality rate of 156.1 versus 99.9 in males and females per 100,000 people, respectively [2]. The risk of developing cancers increased by age in 2007–2017 globally, and the odds of cancer developments in women were higher than men up to 49 years old, while men had a higher incidence of developing cancers between 50 and 80 years old [3].

In order to reduce the cancer-attributable burden and increase the quality of life of patients suffering from cancers, scientists and researchers have developed different therapeutic approaches since decades ago. Despite several investigations in cancer therapy, there is still a long way through to the optimum point. Considering surgery, radiation therapy, and chemotherapy as three pillars of cancer treatments, immunotherapy is the fourth one that offers many promising potentials [4]. The development of cancer immunotherapy in clinical practice initiated from immune checkpoint inhibitors (ICIs), especially ipilimumab, an anti-cytotoxic T lymphocyte-associated antigen 4 (CTLA-4) monoclonal antibody [5]. As a result of the fact that immunotherapeutic strategies modulate the immune system, they might have different efficacies and toxicities compared to conventional chemotherapy or radiotherapy [6]. In addition to common gastrointestinal and hematologic adverse events (AEs) of BsAbs, they might cause some immune-related AEs (irAEs) such as cytokine level rise [7]. In this regard, some strategies have been recommended to reduce irAEs of BsAbs like using premedications (e.g. corticosteroids, antihistamines, antipyretics, and intravenous fluids) and step-up dosing [8]. Also, evaluation of irAEs provides an opportunity to assess the perturbed immune hemostasis which can lead to autoimmunity and has important implication for treatment of immune-mediated diseases [9]. Newly developed BsAbs like AFM13 which target CD30/CD16A antibodies could be associated with less AEs and irAEs [8]. Along with adoptive cellular therapy and vaccination, antibodies are of great interest to scientists worldwide [10, 11]. Targeting two different epitopes, bispecific antibodies (BsAbs) perform new capabilities in diagnostics and therapeutics of cancer. Also, BsAbs might have better efficacy and lower production costs in comparison with a combination of two monoclonal antibodies by targeting two epitopes [12]. By March 2022, the Food and Drug Administration (FDA) approved two BsAbs which are blinatumomab for the treatment of B-cell precursor acute lymphoblastic leukemia (ALL) [13] and amivantamab for the treatment of non-small cell lung cancer (NSCLC) [14]. Many others, more than 50, with different structures, mechanisms of action, targets, and various efficacy and safety are under investigation in clinical trials [15]. The results of which may guide further therapies in the field. Along with hematologic malignancies, many BsAbs are being investigated in solid tumors in clinical trials of phase 1 and 2 to assess the safety and efficacy for further development [15].

To our knowledge, there is no comprehensive study that evaluated the safety and efficacy of BsAbs in solid cancers. Therefore, this systematic review is aimed to determine the safety and efficacy of treatment with BsAb compared to standard therapies such as chemotherapy, radiotherapy, other types of immunotherapies, or combination therapies in adult patients with solid malignancies in controlled clinical trials.

## Methods

This systematic review protocol has been established according to the Preferred Reporting Items for Systematic Reviews and Meta-Analyses Protocol (PRISMA-P) 2015 guideline (S1 Appendix) [16].

### Eligibility criteria

#### Type of participants

Both men and women patients aged >18 years old with solid malignancies of any histologic type in any stage. The diagnosis of cancer should be established based on valid guidelines at the time of the studies. We will also include patients who have been administered combination therapies with BsAbs. Patients who have other comorbidities or metastatic cancers will also be included, while patients with benign tumors will be excluded. Patients with hematologic malignancies such as leukemia or lymphoma will be excluded.

#### Types of interventions

Administration of any BsAbs such as blinatumomab, catumaxomab, duligotuzumab, vanucizumab, cibisatamab, solitomab, istiratumab, navicixizumab, ertumaxomab, zenocutuzumab, flotetuzumab, faricimab, and emicizumab in interventional groups will be included. A list of all included BsAbs is available at S2 Appendix. The included clinical trials should have at least one arm receiving BsAb. We will also include patients who have been administered combination therapies with BsAbs. BsAb in pre-targeted radioimmunotherapy (PRIT) and also bispecific chimeric antigen receptors-T cell (CAR-T cell) therapy will be excluded.

### Types of outcome measures. Primary outcomes

1. Cumulative incidence of any grade AEs in each group
2. Cumulative incidence of severe grade AEs (grade 3–5) in each group
3. Overall survival (OS) (from baseline, i.e., first dose of intervention until death) in each group
4. Progression-free survival (PFS) according to RECIST (response evaluation criteria in solid tumors) 1.1 Criteria [17] (from baseline, i.e. first dose of intervention until disease progression or death) in each group
5. Duration of stable disease according to RECIST 1.1 Criteria [17] in each group
6. Objective response rate (ORR) as the proportion of participants with confirmed complete response (CR) or partial response (PR) according to RECIST 1.1 Criteria [17] in each group
7. Disease control rate as the proportion of participants with confirmed complete response (CR) or partial response (PR) or stable disease according to RECIST 1.1 Criteria [17] in each group

### Secondary outcomes

1. Association between type of cancer and cumulative incidence of AEs
2. Association between type of cancer and cumulative incidence of severe AEs
3. Association between type of cancer and OS
4. Association between type of cancer and PFS
5. Association between type of cancer and ORR
6. Association between type of cancer and duration of stable disease
7. Association between type of cancer and disease control rate
8. Association between stage of cancer and cumulative incidence of AEs
9. Association between stage of cancer and cumulative incidence of severe AEs
10. Association between stage of cancer and OS
11. Association between stage of cancer and PFS
12. Association between stage of cancer and ORR
13. Association between stage of cancer and duration of stable disease
14. Association between stage of cancer and disease control rate

### Type of studies

Peer-reviewed clinical trial studies except for phase Ⅰ trials will be included. Only studies with survival or safety data available will be included in this systematic review.

### Exclusion criteria

1. Studies on conditions other than malignant solid tumors
2. Patients with hematologic malignancies such as leukemia or lymphoma
3. Studies on participants aged ≤ 18 years old
4. Studies that did not assess treatment with BsAb
5. BsAb in PRIT and also bispecific CAR-T cell therapy
6. Studies in which survival measures such as overall response rate, PFS, and duration of stable disease or treatment-related AEs are not presented
7. Clinical trials without control group, phase Ⅰ clinical trials, case reports, pre-print articles, reviews, editorials, meta-analysis, commentary letters, conference proceedings, abstracts, trial protocols, re-analysis of previously published clinical trials, observational studies, retrospective studies, personal opinions, preclinical studies, and book chapters
8. Studies written in languages other than English

### Information sources

#### Electronic search

We will search the following sources:

1. PubMed
2. EMBASE
3. Scopus
4. Web of Science
5. Cochrane Central Register of Controlled Trials (CENTRAL)

Please see S3 Appendix for detailed search strategies. One month before submitting the final manuscript, we will perform an updated search on all mentioned databases. If we identify new studies for inclusion, we will evaluate these and incorporate findings in our review before submission of the final manuscript. We will implement no search filters or limitations on any field such as language, publication type, or time period in searching the electronic databases. We will send results of electronic searches to EndNote X9.0 (Clarivate Analytics, Philadelphia, PA, USA) reference manager, and duplicates will be identified and deleted by using it. Also, duplicates will be identified in the title/abstract screening process.

#### Searching other resources

We will search Clinical-Trials.gov (http://clinicaltrials.gov/) and metaRegister of controlled trials (http://www.isrctn.com/). Also, we will try to identify other potentially eligible trials by conducting backward and forward citation searches from included studies. We will contact corresponding authors for full-text articles, additional data, and unpublished trials.

### Data collection and analysis

#### Selection of studies

Two researches will independently evaluate the title and/or abstract of all retrieved articles based on inclusion and exclusion criteria. Full text of relevant and even potentially relevant articles will be found. Then after, these full texts will be investigated by two reviewers independently to determine the final included studies. Each study which reported abovementioned safety and/or efficacy measures that can be analyzed as continuous measures will be included in meta-analysis. Discrepancies in all stages will be resolved by discussion between the reviewers or consultant with a third review author. An adapted Preferred Reporting Items for Systematic Reviews and Meta-Analyses (PRISMA) flow chart will be prepared (S4 Appendix) [16].

#### Data collection

Two authors will independently abstract characteristics of studies, participants, interventions, and outcomes including first author, year of publication, digital object identifier (DOI) of the article, phase of clinical trial, study design (e.g., parallel or cross-over), funding sources, number of arms, total number of participants, number of participants in each group, mean/median/range of age in each group, number of patients with each gender in each group, number of patients with each cancer type in each group, number of patients with each stage of cancer in each group, number of patients with each race/ethnicity in each group, number of participants completed the study in each group, median follow-up in each group, type of BsAb and its targets, type of control medication, dose and schedule of BsAb and control medication, total number of AEs and irAEs in each group, total number of severe AEs and severe irAEs in each group, total number of DLTs in each group, OS in each group, PFS in each group, ORR in each group, duration of stable disease in each group, and disease control rate in each group [18]. Disagreements between reviewers will be solved by discussion or consultation with a third author. Relevant missing information will be requested from corresponding authors via emailing them.

#### Study quality assessment

Two review authors will independently use The Cochrane Collaboration’s risk of bias tool 2 (RoB2) for assessing the risk of bias and quality assessment [19]. This tool includes bias due to five domains, including randomizations process, deviation from intended interventions, missing outcome data, measurement of the outcome, and selection of the reported result in addition to overall bias [19]. In case of any disagreement between the reviewers, we will resolve it by consultation with a third author. Summary of quality assessment results will be showed in a table.

#### Publication bias

In case of at least ten eligible studies, publication bias will be evaluated visually by drawing a funnel plot [20]. In order to interpret the visual assessment of funnel plots numerically, we will undertake Egger’s test for continuous and dichotomous data [21] in case of no heterogeneity between studies [22]. If there is any amount of heterogeneity, Peter’s test and Egger’s test will be performed for dichotomous and continuous data, respectively [21, 23]. Both Egger’s and Peter’s tests will be used if at least ten studies are included in the study.

### Data synthesis

#### Statistical analysis

Dichotomous data will be expressed as risk ratios (RRs) with 95% confidence intervals (CIs), including cumulative incidence of any grade of AEs and irAEs, severe AEs and severe irAEs, DLTs, ORR, and disease control rate. Standardized/raw mean differences (MD) with 95% CIs will be expressed for continuous data, including OS, PFS, and duration of stable disease. STATA 16 (STATA Corp LLC, TX) software will be used for meta-analysis if applicable. In order to find the source of heterogeneity, we will implement subgroup analysis based on sex, type of cancer, and stage of cancer. Also, meta-regression will be used for age. In addition, the metaprop command of STATA will be used to calculate the frequency of AEs in intervention and control groups.

#### Dealing with zero cells

We will add continuity correction of 0.5 to cells of each one of intervention or control arms that are zero [24]. Furthermore, we will perform sensitivity analysis to compare the effects of this type of continuity correction [25] because some studies have criticized this method due to its effects on meta-analysis results [26, 27].

#### Assessment of heterogeneity

I^2^ index for heterogeneity will be calculated by Q statistics tests for assessment of heterogeneity [28]. According to the Cochrane Handbook for Systematic Reviews of Interventions, the I^2^ level more than 40% is considered significant [28]. As a result, random-effect model meta-analysis will be undertaken if the heterogeneity is more than 40% [18]. Otherwise, fixed-effect meta-analysis will be used.

#### Sensitivity analysis

We will undertake sensitivity analysis to evaluate the effects of continuity correction and roles of funding sources on the effect size when applicable.

#### Confidence in cumulative evidence

We will use the Grading of Recommendations Assessment Development and Evaluation (GRADE) instrument in order to assess the quality of evidence as four levels of high quality, moderate quality, low quality, and very low quality [29].

## Discussion

The systematic review presented in this protocol is in response to our narrative review on the clinical application of BsAbs [30]. We will report the results of the presented systematic review and meta-analysis in accordance to PRISMA statements [31] and PRISMA harm checklist for reporting safety results [32].

The safety and/or efficacy of some other types of immunotherapeutic methods like ICIs [33] and CAR-T cell therapy [34] have been assessed for solid tumors. Also, findings of a systematic review and meta-analysis showed that the combination immunotherapy, especially with different types of ICIs, was associated with a higher rate of AEs and a better efficacy in comparison with monotherapy [35]. The article by Runcie et al. discussed different types of bispecific and trispecific antibodies for tumors [36], while there is still a gap of knowledge on the safety and efficacy of BsAbs which needs a systematic review and meta-analysis to evaluate them comprehensively.

First of all, we will evaluate the safety and efficacy of BsAb in the treatment of solid tumors. Then, we will assess the effects of age, sex, type, and stage of the cancers on survival and toxicity measures by subgroup analysis or meta-regression. The findings of this systematic review can help physicians in clinical practice and can guide researchers to design further studies.

## Supporting information

S1 Appendix PRISMA-P 2015 checklist. (DOCX)

S2 Appendix List of bispecific antibodies which will be included in this study. (DOCX)

S3 Appendix Search strategy for electronic databases. (DOCX)

S4 Appendix PRISMA flow diagram representing the search process. (DOCX)

## List of abbreviations

DALYs: Disability-adjusted life years
ICIs: Immune checkpoint inhibitors
CTLA-4: Cytotoxic T lymphocyte-associated antigen 4
BsAbs: Bispecific antibodies
FDA: Food and Drug Administration
ALL: Acute lymphoblastic leukemia
NSCLC: Non-small cell lung cancer
AEs: Adverse events
irAEs: Immune-related adverse events
PRISMA-P: Preferred Reporting Items for Systematic Reviews and Meta-Analyses Protocol
PRIT: Pre-targeted radioimmunotherapy
CAR-T cell: Chimeric antigen receptors-T cell
DLTs: Dose limiting toxicities
OS: Overall survival
PFS: Progression-free survival
RECIST: Response Evaluation Criteria In Solid Tumors
ORR: Objective response rate
CR: Complete response
PR: Partial response
CENTRAL: Cochrane Central Register of Controlled Trials
PRISMA: Preferred Reporting Items for Systematic Reviews and Meta-Analyses
DOI: Digital Object Identifier
RoB2: Cochrane Collaboration’s risk of bias tool 2
RR: Risk ratios
CI: Confidence intervals
MD: Mean differences
GRADE: Grading of Recommendations Assessment Development and Evaluation
