# Supplementary material for: Clinical safety and efficacy of bispecific antibody in the treatment of solid tumors: A protocol for a systematic review
Source: PLoS One. 2022 Jul 18;17(7):e0271506. doi: 10.1371/journal.pone.0271506 (PMC9292075; doi:10.1371/journal.pone.0271506)
Supplement: S2 Appendix — (DOCX) [file pone.0271506.s002.docx]

**Supplementary information S2 appendix.** List of bispecific antibodies which will be included in this study.

|  | **Bispecific antibody** | **Reference** |
| --- | --- | --- |
| 1 | Blinatumomab | (1) |
| 2 | Catumaxomab |  |
| 3 | MEHD7945A |  |
|  | Duligotuzumab |  |
| 4 | AFM13 |  |
| 5 | AMG110 |  |
| 6 | AMG211 |  |
| 7 | BI836880 |  |
| 8 | BIS-1 |  |
| 9 | CD20Bi |  |
| 10 | DT2219 |  |
| 11 | EGFRBi |  |
| 12 | EGFR-nanocell-paclitaxel |  |
| 13 | EGFR-nanocell-doxorubicin |  |
| 14 | F6–734/hMN14–734 |  |
| 15 | FBTA05 |  |
| 16 | HER2Bi |  |
| 17 | IMCgp100 |  |
| 18 | LY3164530 |  |
| 19 | MCLA-128 |  |
| 20 | MDX-447 |  |
| 21 | MM-111 |  |
| 22 | MM-141 |  |
| 23 | OMP-305B83 |  |
| 24 | RG7802 |  |
|  | RO6958688 |  |
| 25 | RO6874813 |  |
| 26 | TargoMIRs |  |
| 27 | Vanucizumab |  |
| 28 | ZW25 |  |
| 29 | ABT-165 |  |
| 30 | BTCT4465A |  |
| 31 | MGD007 |  |
| 32 | MGD009 |  |
| 33 | REGN1979 |  |
| 34 | RO7082859 |  |
| 35 | ERY972 |  |
| 36 | MCLA-117 |  |
| 37 | MEDI5752 |  |
| 38 | OMP305B83 |  |
| 39 | JNJ-61186372 |  |
| 40 | JNJ-64007957 |  |
| 41 | ABT165 |  |
| 42 | NOV1501 |  |
|  | ABL001 |  |
| 43 | Cerebral EDV |  |
| 44 | KIDEDV |  |
| 45 | XmAb14045 |  |
| 46 | XmAb13676 |  |
| 47 | XmAb18087 |  |
| 48 | XmAb20717 |  |
| 49 | AMG424 |  |
| 50 | GBR1302 |  |
| 51 | GBR1342 |  |
| 52 | ES414 |  |
| 53 | AMG330 |  |
| 54 | BAY2010112 |  |
| 55 | BFCR4350A |  |
| 56 | BI836909 |  |
|  | AMG420 |  |
| 57 | AMG757 |  |
| 58 | MGD006 |  |
| 59 | PF-06671008 |  |
| 60 | MGD013 |  |
| 61 | AFM11 |  |
| 62 | AMV564 |  |
| 63 | DT2219ARL |  |
| 64 | GEM333 |  |
| 65 | IMCnyeso |  |
| 66 | 161,533 |  |
| 67 | ATOR-1015 | (2) |
| 68 | Cibisatamab |  |
|  | RG-7802 |  |
|  | WHO 10636 |  |
|  | WHO10636 |  |
| 69 | MV-BiTEs |  |
| 70 | MEDI-565 |  |
|  | MT111 |  |
| 71 | M701 |  |
|  | Solitomab |  |
|  | MT110 |  |
| 72 | huA33-BsAb |  |
| 73 | EK-02 |  |
| 74 | 16 × 133 BiKE |  |
| 75 | EpCAM16 BiKE |  |
| 76 | XGFR |  |
| 77 | Istiratumab |  |
|  | MM141 |  |
| 78 | GSK3178022 |  |
| 79 | RG7221 |  |
|  | RO5520985 |  |
| 80 | MS133 |  |
| 81 | ER-Met |  |
| 82 | HER-2×CD3 |  |
| 83 | hEx3-scFv-Fc |  |
| 84 | hEx3-scDb-Fc |  |
| 85 | Navicixizumab |  |
| 86 | Ertumaxomab |  |
| 87 | CEA/CD3-bscAb |  |
| 88 | EpCAM16 |  |
| 89 | 1615EpCAM TriKE |  |
| 90 | 1615EpCAM |  |
| 91 | Humanized 3F8 | Clinicaltrials.gov* |
|  | Hu3F8-BsAb |  |
| 92 | MCLA-158 |  |
| 93 | AK104 |  |
| 94 | 10E8.4/iMab |  |
| 95 | MCLA-145 |  |
| 96 | MDX447 |  |
| 97 | LY3434172 |  |
| 98 | PF-06863135 |  |
| 99 | Zenocutuzumab |  |
| 100 | CD3-MUC1 |  |
| 101 | RO7247669 |  |
| 102 | RO7121661 |  |
| 103 | GEM3PSCA |  |
| 104 | rM28 |  |
| 105 | AK112 |  |
| 106 | MBS301 |  |
| 107 | TNB-383B |  |
| 108 | CC-1 |  |
| 109 | MT103 |  |
| 110 | JNJ-64407564 |  |
| 111 | MGD019 |  |
| 112 | GBR 1342 |  |
| 113 | Removab |  |
| 114 | BCD-121 |  |
| 115 | AGEN1223 |  |
| 116 | Flotetuzumab |  |
| 117 | ES101 |  |
| 118 | LY3415244 |  |
| 119 | AK-104 |  |
| 120 | IBI315 |  |
| 121 | 4G7xH22 |  |
| 122 | TG-1801 |  |
| 123 | BCD-147 |  |
| 124 | MGD010 |  |
| 125 | ISB 1302 |  |
| 126 | AMG 160 |  |
| 127 | REGN5459 |  |
| 128 | REGN5458 |  |
| 129 | INBRX-105 |  |
| 130 | MGD014 |  |
| 131 | REGN4018 |  |
| 132 | ERY974 |  |
| 133 | AMG 199 |  |
| 134 | JNJ-67571244 |  |
| 135 | AMG 910 |  |
| 136 | MEDI-538 |  |
| 137 | RG7716 |  |
| 138 | RO6867461 |  |
| 139 | Faricimab |  |
| 140 | PRS-343 |  |
| 141 | GBR 1302 |  |
| 143 | FS118 |  |
| 144 | AMG 757 |  |
| 145 | XmAb®23104 |  |
| 146 | XmAb®20717 |  |
| 147 | XmAb®18087 |  |
| 148 | PRV-3279 |  |
| 149 | AFM24 |  |
| 150 | KN046 |  |
| 151 | KN026 |  |
| 152 | XmAb®22841 |  |
| 153 | APVO436 |  |
| 154 | AMG 330 |  |
| 155 | RO5534262 |  |
|  | Emicizumab |  |
|  | RG6013 |  |
|  | ACE910 |  |
|  | Hemlibra |  |
| 156 | MT112 |  |
| 157 | EMB-01 |  |
|  | FIT-013a |  |
| 158 | ZW49 |  |
| 159 | AMG 509 |  |

*We used search term “bispecific” for searching clinicaltrilas.gov on March 14, 2020. (Search results are available at: <https://clinicaltrials.gov/ct2/results?cond=&term=bispecific&cntry=&state=&city=&dist>=)

**References**

1. Suurs FV, Lub-de Hooge MN, de Vries EGE, de Groot DJA. A review of bispecific antibodies and antibody constructs in oncology and clinical challenges. Pharmacol Ther. 2019;201:103-19.

2. Balibegloo M, Rezaei N. Development and clinical application of bispecific antibody in the treatment of colorectal cancer. Expert Review of Clinical Immunology. 2020;16(7):689-709.
