# Supplementary material for: Clinical safety and efficacy of bispecific antibody in the treatment of solid tumors: A protocol for a systematic review
Source: PLoS One. 2022 Jul 18;17(7):e0271506. doi: 10.1371/journal.pone.0271506 (PMC9292075; doi:10.1371/journal.pone.0271506)
Supplement: S3 Appendix — (DOCX) [file pone.0271506.s003.docx]

**Supplementary information S3 appendix.** Search strategy for electronic databases

**Table 1. Search strategy for PubMed database.**

|  | **Search strategy** |
| --- | --- |
| #1 | (((bispecific*[Title/Abstract] OR “bi-specific*”[Title/Abstract] OR 2specific*[Title/Abstract] OR “2-specific*”[Title/Abstract] OR “two-specific*”[Title/Abstract] OR “double-specific*”[Title/Abstract] OR dualspecific*[Title/Abstract] OR “dual-specific*”[Title/Abstract] OR despecific*[Title/Abstract] OR “de-specific*”[Title/Abstract] OR polyspecific*[Title/Abstract] OR “poly-specific*”[Title/Abstract] OR bifunction*[Title/Abstract] OR “bi-function*”[Title/Abstract] OR “Antibodies, Bispecific”[MeSH Terms]) AND (antibody[Title/Abstract] OR “anti-body”[Title/Abstract] OR Immunoglobulin[Title/Abstract] OR “Immuno-globulin”[Title/Abstract] OR “Immune-globulin”[Title/Abstract] OR IgG[Title/Abstract] OR Ig[Title/Abstract] OR moAb[Title/Abstract] OR mAb[Title/Abstract] OR BiTE[Title/Abstract] OR “T-cell engag*”[Title/Abstract] OR Igs[Title/Abstract] OR mAbs[Title/Abstract] OR antibodies[Title/Abstract] OR “anti-bodies”[Title/Abstract] OR Immunoglobulins[Title/Abstract] OR “Immuno-globulins”[Title/Abstract] OR “Immune-globulins”[Title/Abstract] OR IgGs[Title/Abstract] OR moAbs[Title/Abstract] OR BiTEs[Title/Abstract] OR antibody[MeSH Terms])) OR (blinatumomab[Title/Abstract] OR Catumaxomab[Title/Abstract] OR MEHD7945A[Title/Abstract] OR Duligotuzumab[Title/Abstract] OR AFM13[Title/Abstract] OR AMG110[Title/Abstract] OR AMG211[Title/Abstract] OR BI836880[Title/Abstract] OR “BIS-1”[Title/Abstract] OR CD20Bi[Title/Abstract] OR DT2219[Title/Abstract] OR EGFRBi[Title/Abstract] OR “EGFR-nanocell-paclitaxel”[Title/Abstract] OR “EGFR-nanocell-doxorubicin”[Title/Abstract] OR “F6–734/hMN14–734”[Title/Abstract] OR FBTA05[Title/Abstract] OR HER2Bi[Title/Abstract] OR IMCgp100[Title/Abstract] OR LY3164530[Title/Abstract] OR “MCLA-128”[Title/Abstract] OR “MDX-447”[Title/Abstract] OR “MM-111”[Title/Abstract] OR “MM-141”[Title/Abstract] OR “OMP-305B83”[Title/Abstract] OR RG7802[Title/Abstract] OR RO6958688[Title/Abstract] OR RO6874813[Title/Abstract] OR TargoMIRs[Title/Abstract] OR Vanucizumab[Title/Abstract] OR ZW25[Title/Abstract] OR “ABT-165”[Title/Abstract] OR BTCT4465A[Title/Abstract] OR MGD007[Title/Abstract] OR MGD009[Title/Abstract] OR REGN1979[Title/Abstract] OR RO7082859[Title/Abstract] OR ERY972[Title/Abstract] OR “MCLA-117”[Title/Abstract] OR MEDI5752[Title/Abstract] OR OMP305B83[Title/Abstract] OR “JNJ-61186372”[Title/Abstract] OR “JNJ-64007957”[Title/Abstract] OR ABT165[Title/Abstract] OR NOV1501[Title/Abstract] OR ABL001[Title/Abstract] OR “Cerebral EDV”[Title/Abstract] OR KIDEDV[Title/Abstract] OR XmAb14045[Title/Abstract] OR XmAb13676[Title/Abstract] OR XmAb18087[Title/Abstract] OR XmAb20717[Title/Abstract] OR AMG424[Title/Abstract] OR GBR1302[Title/Abstract] OR GBR1342[Title/Abstract] OR ES414[Title/Abstract] OR AMG330,[Title/Abstract] OR BAY2010112[Title/Abstract] OR BFCR4350A[Title/Abstract] OR BI836909[Title/Abstract] OR AMG420[Title/Abstract] OR AMG757[Title/Abstract] OR MGD006[Title/Abstract] OR “PF-06671008”[Title/Abstract] OR MGD013[Title/Abstract] OR AFM11[Title/Abstract] OR AMV564[Title/Abstract] OR DT2219ARL[Title/Abstract] OR GEM333[Title/Abstract] OR IMCnyeso[Title/Abstract] OR “161,533”[Title/Abstract] OR “ATOR-1015”[Title/Abstract] OR Cibisatamab[Title/Abstract] OR “RG-7802”[Title/Abstract] OR “WHO 10636”[Title/Abstract] OR WHO10636[Title/Abstract] OR “MV-BiTEs”[Title/Abstract] OR “MEDI-565”[Title/Abstract] OR MT111[Title/Abstract] OR M701[Title/Abstract] OR Solitomab[Title/Abstract] OR MT110[Title/Abstract] OR “huA33-BsAb”[Title/Abstract] OR “EK-02”[Title/Abstract] OR “16 × 133 BiKE”[Title/Abstract] OR “EpCAM16 BiKE”[Title/Abstract] OR “XGFR*”[Title/Abstract] OR Istiratumab[Title/Abstract] OR MM141[Title/Abstract] OR GSK3178022[Title/Abstract] OR RG7221[Title/Abstract] OR RO5520985[Title/Abstract] OR MS133[Title/Abstract] OR “ER-Met”[Title/Abstract] OR “HER-2×CD3”[Title/Abstract] OR “hEx3-scFv-Fc”[Title/Abstract] OR “hEx3-scDb-Fc”[Title/Abstract] OR Navicixizumab[Title/Abstract] OR Ertumaxomab[Title/Abstract] OR “CEA/CD3-bscAb”[Title/Abstract] OR EpCAM16[Title/Abstract] OR “1615EpCAM TriKE”[Title/Abstract] OR 1615EpCAM[Title/Abstract] OR “Humanized 3F8”[Title/Abstract] OR “Hu3F8-BsAb”[Title/Abstract] OR “MCLA-158”[Title/Abstract] OR AK104[Title/Abstract] OR “10E8.4/iMab”[Title/Abstract] OR “MCLA-145”[Title/Abstract] OR MDX447[Title/Abstract] OR LY3434172[Title/Abstract] OR “PF-06863135”[Title/Abstract] OR zenocutuzumab[Title/Abstract] OR “CD3-MUC1”[Title/Abstract] OR RO7247669[Title/Abstract] OR RO7121661[Title/Abstract] OR GEM3PSCA[Title/Abstract] OR rM28[Title/Abstract] OR AK112[Title/Abstract] OR MBS301[Title/Abstract] OR “TNB-383B”[Title/Abstract] OR “CC-1”[Title/Abstract] OR MT103[Title/Abstract] OR “JNJ-64407564”[Title/Abstract] OR MGD019[Title/Abstract] OR “GBR 1342”[Title/Abstract] OR removab[Title/Abstract] OR “BCD-121”[Title/Abstract] OR AGEN1223[Title/Abstract] OR Flotetuzumab[Title/Abstract] OR ES101[Title/Abstract] OR LY3415244[Title/Abstract] OR “AK-104”[Title/Abstract] OR IBI315[Title/Abstract] OR “4G7xH22”[Title/Abstract] OR “TG-1801”[Title/Abstract] OR “BCD-147”[Title/Abstract] OR MGD010[Title/Abstract] OR “ISB 1302”[Title/Abstract] OR “AMG 160”[Title/Abstract] OR REGN5459[Title/Abstract] OR REGN5458[Title/Abstract] OR “INBRX-105”[Title/Abstract] OR MGD014[Title/Abstract] OR REGN4018[Title/Abstract] OR ERY974[Title/Abstract] OR “AMG 199”[Title/Abstract] OR “JNJ-67571244”[Title/Abstract] OR “AMG 910”[Title/Abstract] OR “MEDI-538”[Title/Abstract] OR RG7716[Title/Abstract] OR RO6867461[Title/Abstract] OR Faricimab [Title/Abstract] OR “PRS-343”[Title/Abstract] OR “GBR 1302”[Title/Abstract] OR FS118[Title/Abstract] OR “AMG 757”[Title/Abstract] OR “XmAb®23104”[Title/Abstract] OR “XmAb®20717”[Title/Abstract] OR “XmAb®18087”[Title/Abstract] OR “PRV-3279”[Title/Abstract] OR AFM24[Title/Abstract] OR KN046[Title/Abstract] OR KN026[Title/Abstract] OR “XmAb®22841”[Title/Abstract] OR APVO436[Title/Abstract] OR “AMG 330”[Title/Abstract] OR RO5534262[Title/Abstract] OR Emicizumab[Title/Abstract] OR RG6013[Title/Abstract] OR ACE910[Title/Abstract] OR Hemlibra[Title/Abstract] OR MT112[Title/Abstract] OR “EMB-01”[Title/Abstract] OR “FIT-013a”[Title/Abstract] OR ZW49[Title/Abstract] OR “AMG 509”[Title/Abstract] OR Blincyto[Title/Abstract] OR “MT-103”[Title/Abstract] OR “AMG-330”[Title/Abstract] OR “OC-TR”[Title/Abstract] OR “MDX-H210”[Title/Abstract] OR MDXH210[Title/Abstract] OR “5.2-OKT3”[Title/Abstract] OR MDXH447[Title/Abstract] OR MT110 [Title/Abstract] OR Bi20[Title/Abstract])) AND (cancer[Title/Abstract] OR carcinom*[Title/Abstract] OR malignan*[Title/Abstract] OR neoplas*[Title/Abstract] OR tumor[Title/Abstract] OR tumour[Title/Abstract] OR tumors[Title/Abstract] OR cancers[Title/Abstract] OR tumours[Title/Abstract] OR cancer[MeSH Terms]) |
| #2 | clinical[All Fields] OR trial[All Fields] OR trials[All Fields] OR phase[All Fields] OR "intervention study"[All Fields] OR “clinical trials as topic”[MeSH Terms] |
| #3 | #1 AND #2 |

**Table 2. Search strategy for Scopus database.**

|  | **Search strategy** |
| --- | --- |
| #1 | (((TITLE-ABS-KEY(bispecific* OR “bi-specific*” OR 2specific* OR “2-specific*” OR “two-specific*” OR “double-specific*” OR dualspecific* OR “dual-specific*” OR despecific* OR “de-specific*” OR polyspecific* OR “poly-specific*” OR bifunction* OR “bi-function*”) OR INDEXTERMS(bispecific OR “bispecific antibody”)) AND (TITLE-ABS-KEY(antibody OR “anti-body” OR Immunoglobulin OR “Immuno-globulin” OR “Immune-globulin” OR IgG OR Ig OR moAb OR mAb OR BiTE OR “T-cell engag*” OR Igs OR mAbs OR antibodies OR “anti-bodies” OR Immunoglobulins OR “Immuno-globulins” OR “Immune-globulins” OR IgGs OR moAbs OR BiTEs) OR INDEXTERMS(antibody OR Immunoglobulin))) OR (TITLE-ABS-KEY(blinatumomab OR Catumaxomab OR MEHD7945A OR Duligotuzumab OR AFM13 OR AMG110 OR AMG211 OR BI836880 OR “BIS-1” OR CD20Bi OR DT2219 OR EGFRBi OR “EGFR-nanocell-paclitaxel” OR “EGFR-nanocell-doxorubicin” OR “F6–734/hMN14–734” OR FBTA05 OR HER2Bi OR IMCgp100 OR LY3164530 OR “MCLA-128” OR “MDX-447” OR “MM-111” OR “MM-141” OR “OMP-305B83” OR RG7802 OR RO6958688 OR RO6874813 OR TargoMIRs OR Vanucizumab OR ZW25 OR “ABT-165” OR BTCT4465A OR MGD007 OR MGD009 OR REGN1979 OR RO7082859 OR ERY972 OR “MCLA-117” OR MEDI5752 OR OMP305B83 OR “JNJ-61186372” OR “JNJ-64007957” OR ABT165 OR NOV1501 OR ABL001 OR “Cerebral EDV” OR KIDEDV OR XmAb14045 OR XmAb13676 OR XmAb18087 OR XmAb20717 OR AMG424 OR GBR1302 OR GBR1342 OR ES414 OR AMG330, OR BAY2010112 OR BFCR4350A OR BI836909 OR AMG420 OR AMG757 OR MGD006 OR “PF-06671008” OR MGD013 OR AFM11 OR AMV564 OR DT2219ARL OR GEM333 OR IMCnyeso OR “161,533” OR “ATOR-1015” OR Cibisatamab OR “RG-7802” OR “WHO 10636” OR WHO10636 OR “MV-BiTEs” OR “MEDI-565” OR MT111 OR M701 OR Solitomab OR MT110 OR “huA33-BsAb” OR “EK-02” OR “16 × 133 BiKE” OR “EpCAM16 BiKE” OR “XGFR*” OR Istiratumab OR MM141 OR GSK3178022 OR RG7221 OR RO5520985 OR MS133 OR “ER-Met” OR “HER-2×CD3” OR “hEx3-scFv-Fc” OR “hEx3-scDb-Fc” OR Navicixizumab OR Ertumaxomab OR “CEA/CD3-bscAb” OR EpCAM16 OR “1615EpCAM TriKE” OR 1615EpCAM OR “Humanized 3F8” OR “Hu3F8-BsAb” OR “MCLA-158” OR AK104 OR “10E8.4/iMab” OR “MCLA-145” OR MDX447 OR LY3434172 OR “PF-06863135” OR zenocutuzumab OR “CD3-MUC1” OR RO7247669 OR RO7121661 OR GEM3PSCA OR rM28 OR AK112 OR MBS301 OR “TNB-383B” OR “CC-1” OR MT103 OR “JNJ-64407564” OR MGD019 OR “GBR 1342” OR removab OR “BCD-121” OR AGEN1223 OR Flotetuzumab OR ES101 OR LY3415244 OR “AK-104” OR IBI315 OR “4G7xH22” OR “TG-1801” OR “BCD-147” OR MGD010 OR “ISB 1302” OR “AMG 160” OR REGN5459 OR REGN5458 OR “INBRX-105” OR MGD014 OR REGN4018 OR ERY974 OR “AMG 199” OR “JNJ-67571244” OR “AMG 910” OR “MEDI-538” OR RG7716 OR RO6867461 OR Faricimab OR “PRS-343” OR “GBR 1302” OR FS118 OR “AMG 757” OR “XmAb®23104” OR “XmAb®20717” OR “XmAb®18087” OR “PRV-3279” OR AFM24 OR KN046 OR KN026 OR “XmAb®22841” OR APVO436 OR “AMG 330” OR RO5534262 OR Emicizumab OR RG6013 OR ACE910 OR Hemlibra OR MT112 OR “EMB-01” OR “FIT-013a” OR ZW49 OR “AMG 509” OR Blincyto OR “MT-103” OR “AMG-330” OR “OC-TR” OR “MDX-H210” OR MDXH210 OR “5.2-OKT3” OR MDXH447 OR MT110 OR Bi20))) AND (TITLE-ABS-KEY(cancer OR carcinom* OR malignan* OR neoplas* OR tumor OR tumour OR tumors OR cancers OR tumours) OR INDEXTERMS(cancer)) |
| #2 | ALL(clinical OR trial OR trials OR phase OR "intervention study") OR (INDEXTERMS(“clinical trials as topic”) OR (“clinical trial”)) |
| #3 | #1 AND #2 |

**Table 3. Search strategy for Web of Science database.**

|  | **Search strategy** |
| --- | --- |
| #1 | (((TS=(bispecific* OR “bi-specific*” OR 2specific* OR “2-specific*” OR “two-specific*” OR “double-specific*” OR dualspecific* OR “dual-specific*” OR despecific* OR “de-specific*” OR polyspecific* OR “poly-specific*” OR bifunction* OR “bi-function*”)) AND (TS=(antibody OR “anti-body” OR Immunoglobulin OR “Immuno-globulin” OR “Immune-globulin” OR IgG OR Ig OR moAb OR mAb OR BiTE OR “T-cell engag*” OR Igs OR mAbs OR antibodies OR “anti-bodies” OR Immunoglobulins OR “Immuno-globulins” OR “Immune-globulins” OR IgGs OR moAbs OR BiTEs))) OR (TS=(blinatumomab OR Catumaxomab OR MEHD7945A OR Duligotuzumab OR AFM13 OR AMG110 OR AMG211 OR BI836880 OR “BIS-1” OR CD20Bi OR DT2219 OR EGFRBi OR “EGFR-nanocell-paclitaxel” OR “EGFR-nanocell-doxorubicin” OR “F6–734/hMN14–734” OR FBTA05 OR HER2Bi OR IMCgp100 OR LY3164530 OR “MCLA-128” OR “MDX-447” OR “MM-111” OR “MM-141” OR “OMP-305B83” OR RG7802 OR RO6958688 OR RO6874813 OR TargoMIRs OR Vanucizumab OR ZW25 OR “ABT-165” OR BTCT4465A OR MGD007 OR MGD009 OR REGN1979 OR RO7082859 OR ERY972 OR “MCLA-117” OR MEDI5752 OR OMP305B83 OR “JNJ-61186372” OR “JNJ-64007957” OR ABT165 OR NOV1501 OR ABL001 OR “Cerebral EDV” OR KIDEDV OR XmAb14045 OR XmAb13676 OR XmAb18087 OR XmAb20717 OR AMG424 OR GBR1302 OR GBR1342 OR ES414 OR AMG330, OR BAY2010112 OR BFCR4350A OR BI836909 OR AMG420 OR AMG757 OR MGD006 OR “PF-06671008” OR MGD013 OR AFM11 OR AMV564 OR DT2219ARL OR GEM333 OR IMCnyeso OR “161,533” OR “ATOR-1015” OR Cibisatamab OR “RG-7802” OR “WHO 10636” OR WHO10636 OR “MV-BiTEs” OR “MEDI-565” OR MT111 OR M701 OR Solitomab OR MT110 OR “huA33-BsAb” OR “EK-02” OR “16 × 133 BiKE” OR “EpCAM16 BiKE” OR “XGFR*” OR Istiratumab OR MM141 OR GSK3178022 OR RG7221 OR RO5520985 OR MS133 OR “ER-Met” OR “HER-2×CD3” OR “hEx3-scFv-Fc” OR “hEx3-scDb-Fc” OR Navicixizumab OR Ertumaxomab OR “CEA/CD3-bscAb” OR EpCAM16 OR “1615EpCAM TriKE” OR 1615EpCAM OR “Humanized 3F8” OR “Hu3F8-BsAb” OR “MCLA-158” OR AK104 OR “10E8.4/iMab” OR “MCLA-145” OR MDX447 OR LY3434172 OR “PF-06863135” OR zenocutuzumab OR “CD3-MUC1” OR RO7247669 OR RO7121661 OR GEM3PSCA OR rM28 OR AK112 OR MBS301 OR “TNB-383B” OR “CC-1” OR MT103 OR “JNJ-64407564” OR MGD019 OR “GBR 1342” OR removab OR “BCD-121” OR AGEN1223 OR Flotetuzumab OR ES101 OR LY3415244 OR “AK-104” OR IBI315 OR “4G7xH22” OR “TG-1801” OR “BCD-147” OR MGD010 OR “ISB 1302” OR “AMG 160” OR REGN5459 OR REGN5458 OR “INBRX-105” OR MGD014 OR REGN4018 OR ERY974 OR “AMG 199” OR “JNJ-67571244” OR “AMG 910” OR “MEDI-538” OR RG7716 OR RO6867461 OR Faricimab OR “PRS-343” OR “GBR 1302” OR FS118 OR “AMG 757” OR “XmAb®23104” OR “XmAb®20717” OR “XmAb®18087” OR “PRV-3279” OR AFM24 OR KN046 OR KN026 OR “XmAb®22841” OR APVO436 OR “AMG 330” OR RO5534262 OR Emicizumab OR RG6013 OR ACE910 OR Hemlibra OR MT112 OR “EMB-01” OR “FIT-013a” OR ZW49 OR “AMG 509” OR Blincyto OR “MT-103” OR “AMG-330” OR “OC-TR” OR “MDX-H210” OR MDXH210 OR “5.2-OKT3” OR MDXH447 OR MT110 OR Bi20))) AND (TS=(cancer OR carcinom* OR malignan* OR neoplas* OR tumor OR tumour OR tumors OR cancers OR tumours)) |
| #2 | ALL=(clinical OR trial OR trials OR phase OR "intervention study") |
| #3 | #1 AND #2 |

**Table 4. Search strategy for EMBASE database.**

|  | **Search strategy** |
| --- | --- |
| #1 | ((((bispecific* OR “bi-specific*” OR 2specific* OR “2-specific*” OR “two-specific*” OR “double-specific*” OR dualspecific* OR “dual-specific*” OR despecific* OR “de-specific*” OR polyspecific* OR “poly-specific*” OR bifunction* OR “bi-function*”):ti,ab,kw OR “bispecific antibody”:exp) AND ((antibody OR “anti-body” OR Immunoglobulin OR “Immuno-globulin” OR “Immune-globulin” OR IgG OR Ig OR moAb OR mAb OR BiTE OR “T-cell engag*” OR Igs OR mAbs OR antibodies OR “anti-bodies” OR Immunoglobulins OR “Immuno-globulins” OR “Immune-globulins” OR IgGs OR moAbs OR BiTEs):ti,ab,kw OR (antibody OR immunoglobulin):exp)) OR ((blinatumomab OR Catumaxomab OR MEHD7945A OR Duligotuzumab OR AFM13 OR AMG110 OR AMG211 OR BI836880 OR “BIS-1” OR CD20Bi OR DT2219 OR EGFRBi OR “EGFR-nanocell-paclitaxel” OR “EGFR-nanocell-doxorubicin” OR “F6–734/hMN14–734” OR FBTA05 OR HER2Bi OR IMCgp100 OR LY3164530 OR “MCLA-128” OR “MDX-447” OR “MM-111” OR “MM-141” OR “OMP-305B83” OR RG7802 OR RO6958688 OR RO6874813 OR TargoMIRs OR Vanucizumab OR ZW25 OR “ABT-165” OR BTCT4465A OR MGD007 OR MGD009 OR REGN1979 OR RO7082859 OR ERY972 OR “MCLA-117” OR MEDI5752 OR OMP305B83 OR “JNJ-61186372” OR “JNJ-64007957” OR ABT165 OR NOV1501 OR ABL001 OR “Cerebral EDV” OR KIDEDV OR XmAb14045 OR XmAb13676 OR XmAb18087 OR XmAb20717 OR AMG424 OR GBR1302 OR GBR1342 OR ES414 OR AMG330, OR BAY2010112 OR BFCR4350A OR BI836909 OR AMG420 OR AMG757 OR MGD006 OR “PF-06671008” OR MGD013 OR AFM11 OR AMV564 OR DT2219ARL OR GEM333 OR IMCnyeso OR “161,533” OR “ATOR-1015” OR Cibisatamab OR “RG-7802” OR “WHO 10636” OR WHO10636 OR “MV-BiTEs” OR “MEDI-565” OR MT111 OR M701 OR Solitomab OR MT110 OR “huA33-BsAb” OR “EK-02” OR “16 × 133 BiKE” OR “EpCAM16 BiKE” OR “XGFR*” OR Istiratumab OR MM141 OR GSK3178022 OR RG7221 OR RO5520985 OR MS133 OR “ER-Met” OR “HER-2×CD3” OR “hEx3-scFv-Fc” OR “hEx3-scDb-Fc” OR Navicixizumab OR Ertumaxomab OR “CEA/CD3-bscAb” OR EpCAM16 OR “1615EpCAM TriKE” OR 1615EpCAM OR “Humanized 3F8” OR “Hu3F8-BsAb” OR “MCLA-158” OR AK104 OR “10E8.4/iMab” OR “MCLA-145” OR MDX447 OR LY3434172 OR “PF-06863135” OR zenocutuzumab OR “CD3-MUC1” OR RO7247669 OR RO7121661 OR GEM3PSCA OR rM28 OR AK112 OR MBS301 OR “TNB-383B” OR “CC-1” OR MT103 OR “JNJ-64407564” OR MGD019 OR “GBR 1342” OR removab OR “BCD-121” OR AGEN1223 OR Flotetuzumab OR ES101 OR LY3415244 OR “AK-104” OR IBI315 OR “4G7xH22” OR “TG-1801” OR “BCD-147” OR MGD010 OR “ISB 1302” OR “AMG 160” OR REGN5459 OR REGN5458 OR “INBRX-105” OR MGD014 OR REGN4018 OR ERY974 OR “AMG 199” OR “JNJ-67571244” OR “AMG 910” OR “MEDI-538” OR RG7716 OR RO6867461 OR Faricimab OR “PRS-343” OR “GBR 1302” OR FS118 OR “AMG 757” OR “XmAb®23104” OR “XmAb®20717” OR “XmAb®18087” OR “PRV-3279” OR AFM24 OR KN046 OR KN026 OR “XmAb®22841” OR APVO436 OR “AMG 330” OR RO5534262 OR Emicizumab OR RG6013 OR ACE910 OR Hemlibra OR MT112 OR “EMB-01” OR “FIT-013a” OR ZW49 OR “AMG 509” OR Blincyto OR “MT-103” OR “AMG-330” OR “OC-TR” OR “MDX-H210” OR MDXH210 OR “5.2-OKT3” OR MDXH447 OR MT110 OR Bi20):ti,ab,kw)) AND ((cancer OR carcinom* OR malignan* OR neoplas* OR tumor OR tumour OR tumors OR cancers OR tumours):ti,ab,kw OR cancer:exp) |
| #2 | clinical OR trial OR trials OR phase OR "intervention study" OR (“clinical trials as topic” OR “clinical trial”):exp |
| #3 | #1 AND #2 |

**Table 5. Search strategy for the Cochrane Central Register of Controlled Trials (CENTRAL) database.**

|  | **Search strategy** |
| --- | --- |
| #1 | (((bispecific*:ti,ab,kw OR "bi specific*":ti,ab,kw OR 2specific*:ti,ab,kw OR "2 specific*":ti,ab,kw OR "two specific*":ti,ab,kw OR "double specific*":ti,ab,kw OR dualspecific*:ti,ab,kw OR "dual specific*":ti,ab,kw OR despecific*:ti,ab,kw OR "de specific*":ti,ab,kw OR polyspecific*:ti,ab,kw OR "poly specific*":ti,ab,kw OR bifunction*:ti,ab,kw OR "bi function*":ti,ab,kw OR [mh "antibodies, bispecific"]) AND (antibody:ti,ab,kw OR "anti-body":ti,ab,kw OR Immunoglobulin:ti,ab,kw OR "Immuno-globulin":ti,ab,kw OR "Immune-globulin":ti,ab,kw OR IgG:ti,ab,kw OR Ig:ti,ab,kw OR moAb:ti,ab,kw OR mAb:ti,ab,kw OR BiTE:ti,ab,kw OR "T-cell engag*":ti,ab,kw OR Igs:ti,ab,kw OR mAbs:ti,ab,kw OR antibodies:ti,ab,kw OR "anti-bodies":ti,ab,kw OR Immunoglobulins:ti,ab,kw OR "Immuno-globulins":ti,ab,kw OR "Immune-globulins":ti,ab,kw OR IgGs:ti,ab,kw OR moAbs:ti,ab,kw OR BiTEs:ti,ab,kw OR [mh antibodies])) OR (blinatumomab:ti,ab,kw OR Catumaxomab:ti,ab,kw OR MEHD7945A:ti,ab,kw OR Duligotuzumab:ti,ab,kw OR AFM13:ti,ab,kw OR AMG110:ti,ab,kw OR AMG211:ti,ab,kw OR BI836880:ti,ab,kw OR "BIS-1":ti,ab,kw OR CD20Bi:ti,ab,kw OR DT2219:ti,ab,kw OR EGFRBi:ti,ab,kw OR "EGFR-nanocell-paclitaxel":ti,ab,kw OR "EGFR-nanocell-doxorubicin":ti,ab,kw OR "F6–734/hMN14–734":ti,ab,kw OR FBTA05:ti,ab,kw OR HER2Bi:ti,ab,kw OR IMCgp100:ti,ab,kw OR LY3164530:ti,ab,kw OR "MCLA-128":ti,ab,kw OR "MDX-447":ti,ab,kw OR "MM-111":ti,ab,kw OR "MM-141":ti,ab,kw OR "OMP-305B83":ti,ab,kw OR RG7802:ti,ab,kw OR RO6958688:ti,ab,kw OR RO6874813:ti,ab,kw OR TargoMIRs:ti,ab,kw OR Vanucizumab:ti,ab,kw OR ZW25:ti,ab,kw OR "ABT-165":ti,ab,kw OR BTCT4465A:ti,ab,kw OR MGD007:ti,ab,kw OR MGD009:ti,ab,kw OR REGN1979:ti,ab,kw OR RO7082859:ti,ab,kw OR ERY972:ti,ab,kw OR "MCLA-117":ti,ab,kw OR MEDI5752:ti,ab,kw OR OMP305B83:ti,ab,kw OR "JNJ-61186372":ti,ab,kw OR "JNJ-64007957":ti,ab,kw OR ABT165:ti,ab,kw OR NOV1501:ti,ab,kw OR ABL001:ti,ab,kw OR "Cerebral EDV":ti,ab,kw OR KIDEDV:ti,ab,kw OR XmAb14045:ti,ab,kw OR XmAb13676:ti,ab,kw OR XmAb18087:ti,ab,kw OR XmAb20717:ti,ab,kw OR AMG424:ti,ab,kw OR GBR1302:ti,ab,kw OR GBR1342:ti,ab,kw OR ES414:ti,ab,kw OR AMG330,:ti,ab,kw OR BAY2010112:ti,ab,kw OR BFCR4350A:ti,ab,kw OR BI836909:ti,ab,kw OR AMG420:ti,ab,kw OR AMG757:ti,ab,kw OR MGD006:ti,ab,kw OR "PF-06671008":ti,ab,kw OR MGD013:ti,ab,kw OR AFM11:ti,ab,kw OR AMV564:ti,ab,kw OR DT2219ARL:ti,ab,kw OR GEM333:ti,ab,kw OR IMCnyeso:ti,ab,kw OR "161,533":ti,ab,kw OR "ATOR-1015":ti,ab,kw OR Cibisatamab:ti,ab,kw OR "RG-7802":ti,ab,kw OR "WHO 10636":ti,ab,kw OR WHO10636:ti,ab,kw OR "MV-BiTEs":ti,ab,kw OR "MEDI-565":ti,ab,kw OR MT111:ti,ab,kw OR M701:ti,ab,kw OR Solitomab:ti,ab,kw OR MT110:ti,ab,kw OR "huA33-BsAb":ti,ab,kw OR "EK-02":ti,ab,kw OR "16 × 133 BiKE":ti,ab,kw OR "EpCAM16 BiKE":ti,ab,kw OR "XGFR*":ti,ab,kw OR Istiratumab:ti,ab,kw OR MM141:ti,ab,kw OR GSK3178022:ti,ab,kw OR RG7221:ti,ab,kw OR RO5520985:ti,ab,kw OR MS133:ti,ab,kw OR "ER-Met":ti,ab,kw OR "HER-2×CD3":ti,ab,kw OR "hEx3-scFv-Fc":ti,ab,kw OR "hEx3-scDb-Fc":ti,ab,kw OR Navicixizumab:ti,ab,kw OR Ertumaxomab:ti,ab,kw OR "CEA/CD3-bscAb":ti,ab,kw OR EpCAM16:ti,ab,kw OR "1615EpCAM TriKE":ti,ab,kw OR 1615EpCAM:ti,ab,kw OR "Humanized 3F8":ti,ab,kw OR "Hu3F8-BsAb":ti,ab,kw OR "MCLA-158":ti,ab,kw OR AK104:ti,ab,kw OR "10E8.4/iMab":ti,ab,kw OR "MCLA-145":ti,ab,kw OR MDX447:ti,ab,kw OR LY3434172:ti,ab,kw OR "PF-06863135":ti,ab,kw OR zenocutuzumab:ti,ab,kw OR "CD3-MUC1":ti,ab,kw OR RO7247669:ti,ab,kw OR RO7121661:ti,ab,kw OR GEM3PSCA:ti,ab,kw OR rM28:ti,ab,kw OR AK112:ti,ab,kw OR MBS301:ti,ab,kw OR "TNB-383B":ti,ab,kw OR "CC-1":ti,ab,kw OR MT103:ti,ab,kw OR "JNJ-64407564":ti,ab,kw OR MGD019:ti,ab,kw OR "GBR 1342":ti,ab,kw OR removab:ti,ab,kw OR "BCD-121":ti,ab,kw OR AGEN1223:ti,ab,kw OR Flotetuzumab:ti,ab,kw OR ES101:ti,ab,kw OR LY3415244:ti,ab,kw OR "AK-104":ti,ab,kw OR IBI315:ti,ab,kw OR "4G7xH22":ti,ab,kw OR "TG-1801":ti,ab,kw OR "BCD-147":ti,ab,kw OR MGD010:ti,ab,kw OR "ISB 1302":ti,ab,kw OR "AMG 160":ti,ab,kw OR REGN5459:ti,ab,kw OR REGN5458:ti,ab,kw OR "INBRX-105":ti,ab,kw OR MGD014:ti,ab,kw OR REGN4018:ti,ab,kw OR ERY974:ti,ab,kw OR "AMG 199":ti,ab,kw OR "JNJ-67571244":ti,ab,kw OR "AMG 910":ti,ab,kw OR "MEDI-538":ti,ab,kw OR RG7716:ti,ab,kw OR RO6867461:ti,ab,kw OR Faricimab:ti,ab,kw OR "PRS-343":ti,ab,kw OR "GBR 1302":ti,ab,kw OR FS118:ti,ab,kw OR "AMG 757":ti,ab,kw OR "XmAb®23104":ti,ab,kw OR "XmAb®20717":ti,ab,kw OR "XmAb®18087":ti,ab,kw OR "PRV-3279":ti,ab,kw OR AFM24:ti,ab,kw OR KN046:ti,ab,kw OR KN026:ti,ab,kw OR "XmAb®22841":ti,ab,kw OR APVO436:ti,ab,kw OR "AMG 330":ti,ab,kw OR RO5534262:ti,ab,kw OR Emicizumab:ti,ab,kw OR RG6013:ti,ab,kw OR ACE910:ti,ab,kw OR Hemlibra:ti,ab,kw OR MT112:ti,ab,kw OR "EMB-01":ti,ab,kw OR "FIT-013a":ti,ab,kw OR ZW49:ti,ab,kw OR "AMG 509":ti,ab,kw OR Blincyto:ti,ab,kw OR "MT-103":ti,ab,kw OR "AMG-330":ti,ab,kw OR "OC-TR":ti,ab,kw OR "MDX-H210":ti,ab,kw OR MDXH210:ti,ab,kw OR "5.2-OKT3":ti,ab,kw OR MDXH447:ti,ab,kw OR MT110:ti,ab,kw OR Bi20:ti,ab,kw)) AND (cancer:ti,ab,kw OR carcinom*:ti,ab,kw OR malignan*:ti,ab,kw OR neoplas*:ti,ab,kw OR tumor:ti,ab,kw OR tumour:ti,ab,kw OR tumors:ti,ab,kw OR cancers:ti,ab,kw OR tumours:ti,ab,kw OR [mh cancer]) |
| #2 | clinical OR trial OR trials OR phase OR "intervention study" OR [mh "clinical trials as topic"] OR [mh "clinical trial"] |
| #3 | #1 AND #2 |
