# Supplementary material for: Clinical safety and efficacy of bispecific antibody in the treatment of solid tumors: A protocol for a systematic review
Source: PLoS One. 2022 Jul 18;17(7):e0271506. doi: 10.1371/journal.pone.0271506 (PMC9292075; doi:10.1371/journal.pone.0271506)
Supplement: S4 Appendix — (DOCX) [file pone.0271506.s004.docx]

**Supplementary information S4 appendix.** PRISMA flow diagram representing the search process.

**Identification of studies via databases and registers**

Records removed *before screening*:

Duplicate records removed (n = )

Records marked as ineligible by automation tools (n = )

Records removed for other reasons (n = )

Records identified from:

Databases (n = )

Registers (n = )

**Identification**

Records screened

(n = )

Records excluded

(n = )

Reports sought for retrieval

(n = )

Reports not retrieved

(n = )

**Screening**

Reports assessed for eligibility

(n = )

Reports excluded:

Reason 1 (n = )

Reason 2 (n = )

Reason 3 (n = )

etc.

Studies included in review

(n = )

Reports of included studies

(n = )

**Included**

*From:*  Page MJ, McKenzie JE, Bossuyt PM, Boutron I, Hoffmann TC, Mulrow CD, et al. The PRISMA 2020 statement: an updated guideline for reporting systematic reviews. BMJ 2021;372:n71. doi: 10.1136/bmj.n71

For more information, visit: <http://www.prisma-statement.org/>
